# Supplementary figures and images for: Structural determinants of rotavirus proteolytic activation
Source: PLoS Pathog. 2025 Aug 12;21(8):e1013063. doi: 10.1371/journal.ppat.1013063 (PMC12364327; doi:10.1371/journal.ppat.1013063)

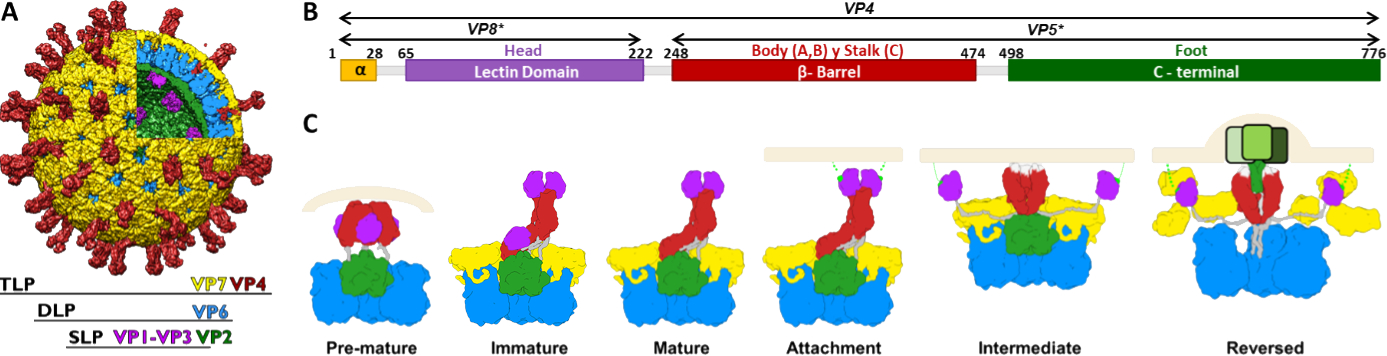

Supplement: S1 Fig — (A). Representation of the RVA viral particle. The different protein layers ensembled and their structural proteins are indicated with the colour code. (B). Schematic representation of the structure and domain organization of VP4. The atomic structure (PDB: 4V7Q) is represented showing each of the monomers that forms the RV spike (VPA-A, VP4-B, VP4-C) and the names of its domains. The panel represent the primary structure of the spike indicating the different domains: α (yellow), lectin (magenta), β barrel (red), and C-terminal (green) domains. The VP4 proteolytic products (VP5* and VP8*) and domains are labelled. Residues delimiting domains and trypsin cleavage sites are indicated. (C). Structural transition of the rotavirus spike during the infectious cycle. Proteins are coloured as indicated: VP6 in blue, VP7 in yellow, VP4/VP5* foot in green, VP4/VP5* β-barrel in red, VP4/VP8* lectin domain in magenta, and loops in grey. During the last stages of the morphogenesis in the endosome, the full-length VP4 monomers in the pre-mature TLP form a flexible 3-fold symmetry structure which carry out a conformational change into the upright structure found in the immature TLP. In this immature spike, two VP4 subunits assemble forming the body and head of the spike (A and B chains) joined by loops (gray). The third VP4 subunit folds to for the stalk with a β-barrel and a lectin domain (C chain). Trypsin proteolysis cleaves the VP4 chains into VP5* and VP8* subproducts. The trypsinization of the α3-β14 loop in three residues, R231, R241 and R247, leads to the loss of the segment 232–247 in the VP4A-B chains and the loss of the lectin domain in VP4C in the mature spike. The activated spike a to the host cell through the interaction of VP8* lectin domains (attachment) with surface glycans (light green) of the cell membrane. This interaction precedes the conformational change in which the lectin domains separate and expose the hydrophobic loops resulting in an intermediate confor [file ppat.1013063.s001.tif]

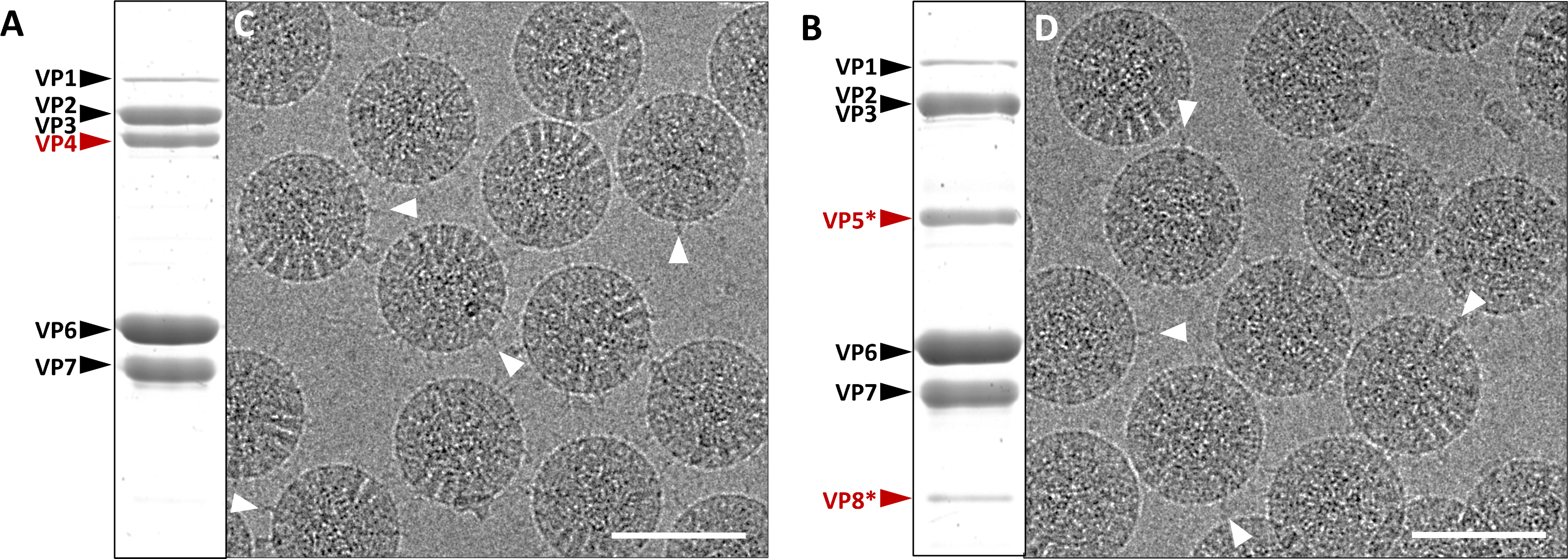

Supplement: S2 Fig — (A, B) Coomassie blue-stained SDS-PAGE of purified TLP, cultured in the absence (A) or presence (B) of trypsin. The positions of RV structural proteins (VPs) are indicated. (C, D) Cryo-electron micrographs of NTR- (C) and TR-TLP (D). The position of some spikes projected from the surface of the particles is indicated with white arrowheads. The bar represents 100 nm. (TIF) [file ppat.1013063.s002.tif]

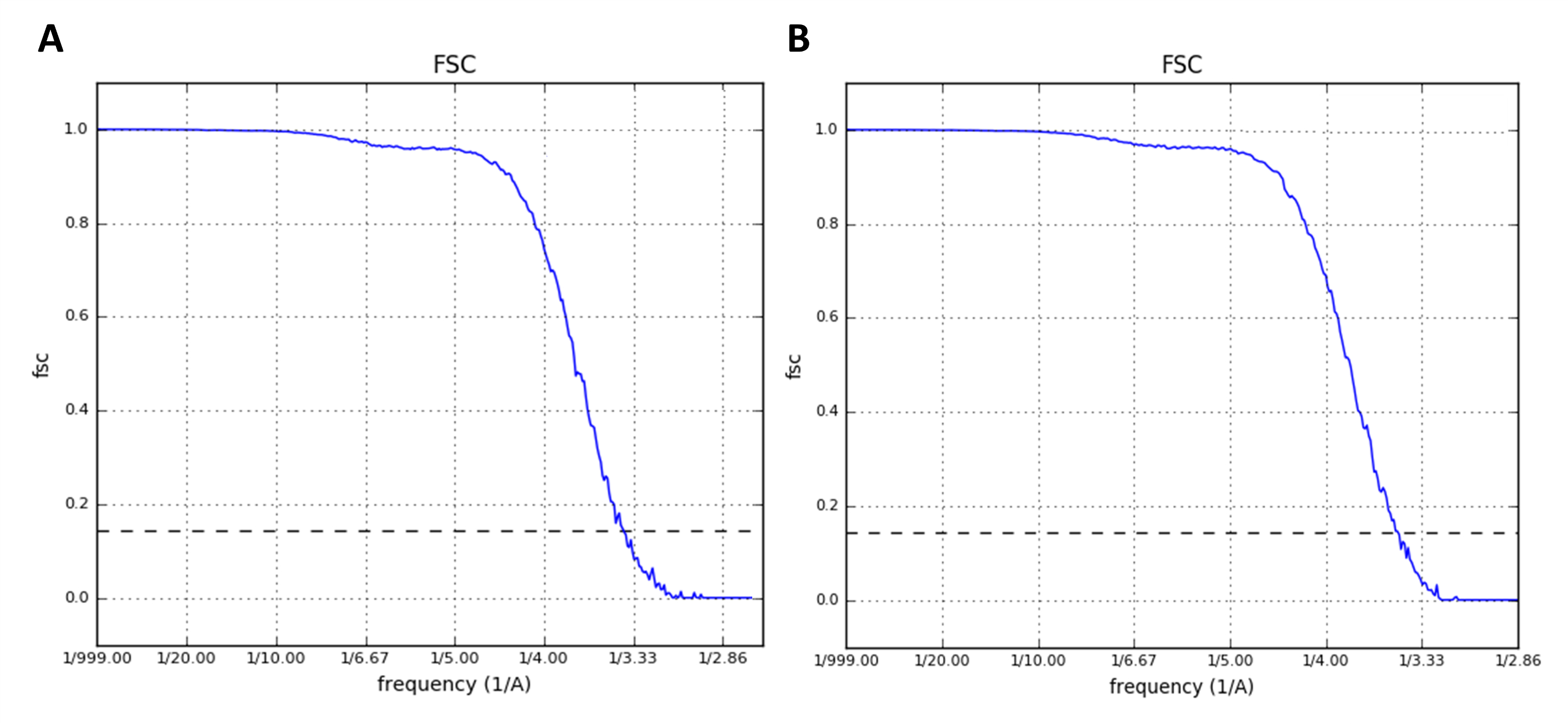

Supplement: S3 Fig — The resolution values of the NTR-TLP, 3.40 Å, and TR-TLP, 3.48 Å, are based on the FSC criterion at 0.143. (TIF) [file ppat.1013063.s003.tif]

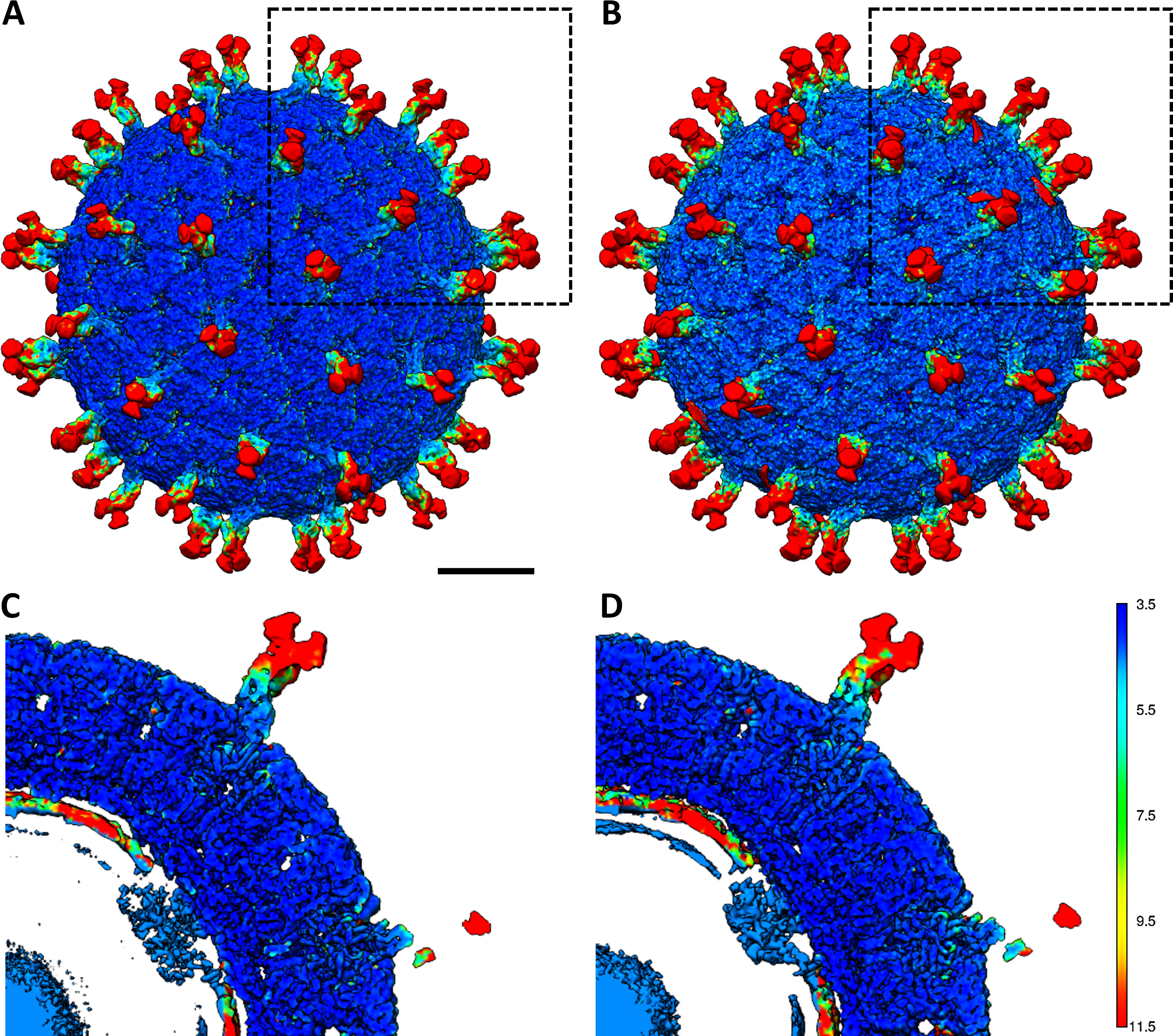

Supplement: S4 Fig — (A-B) Representation of the 3D maps of the NTR- (A) and TR-TLP (B) particle viewed along the icosahedral axis of symmetry 2. The densities observed in panels C and D are indicated with a dashed square. (C-D) Close view of NTR- (C) and TR-TLP (D) cross sections of each 3D map. The sections are parallel but offset 14.7Å from the central section of the maps. The surfaces are coloured according to the local resolution calculated for each 3DR. The colour code is shown with the corresponding resolutions in Å. Densities are contoured at 2σ above the mean. Scale bar represents 100 Å. (TIF) [file ppat.1013063.s004.tif]

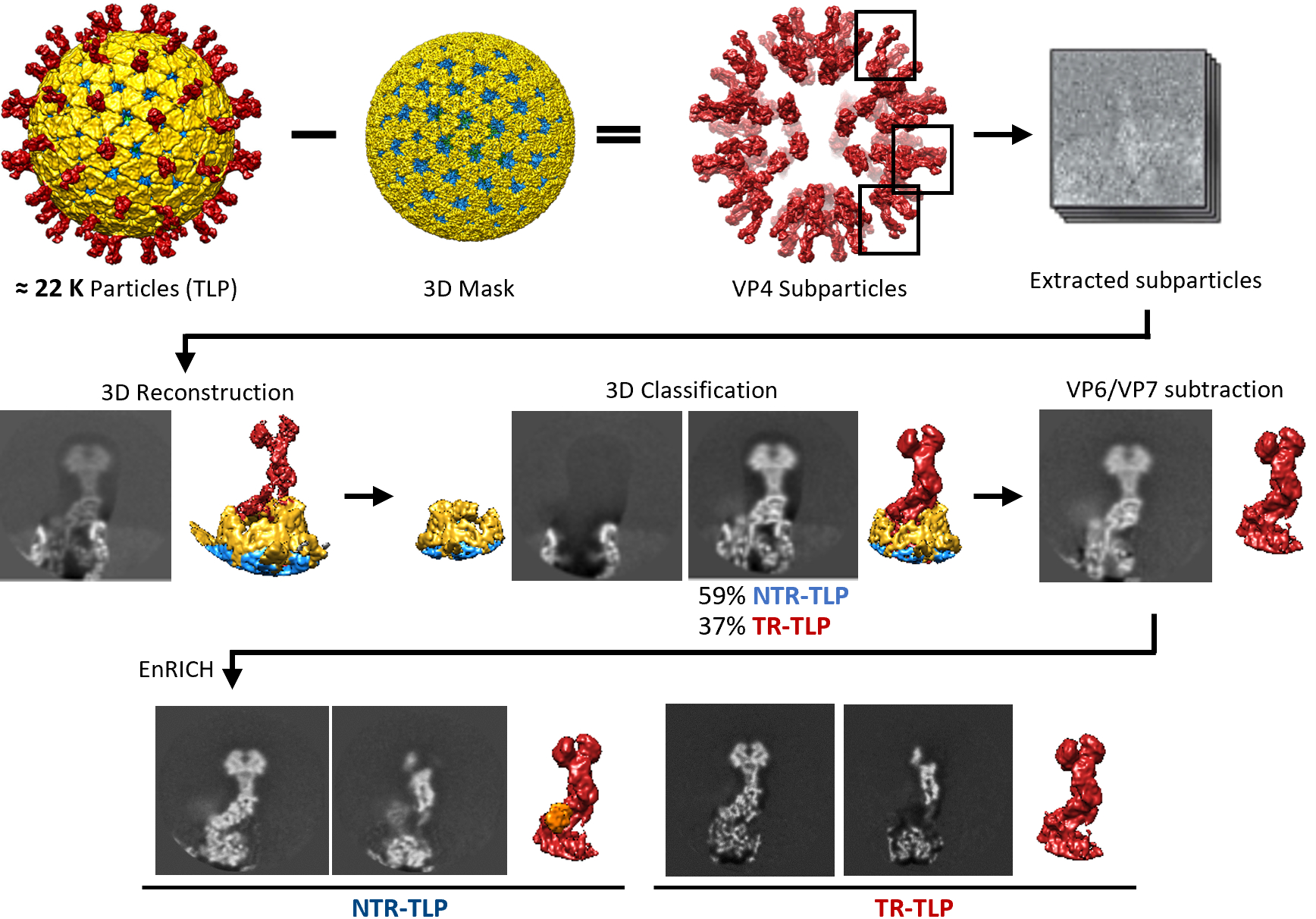

Supplement: S5 Fig — Firstly, we used the localized reconstruction method [43]: the VP2, VP6 and VP7 layers were subtracted using the corresponding TLP maps and a mask that encompasses these three layers. Subsequently, the spikes from all positions were extracted from the calculated difference images and treated as individual particles for their 3D classification, refinement and reconstruction. A 3D classification separated the positions occupied and not occupied by spikes, with an occupancy level of 59 and 37% for NTR- and TR-TLP, in each case. The unoccupied 3DR were used to subtract the VP6 and VP7 signal from the spike-occupied subparticles. Finally, the EnRICH method was applied [46] to obtain aligned subparticles whose 3D reconstructions showed a significant increase in their local resolutions, contrast and signal-to-noise ratio in the stalk, body, and head regions for both spikes. (TIF) [file ppat.1013063.s005.tif]

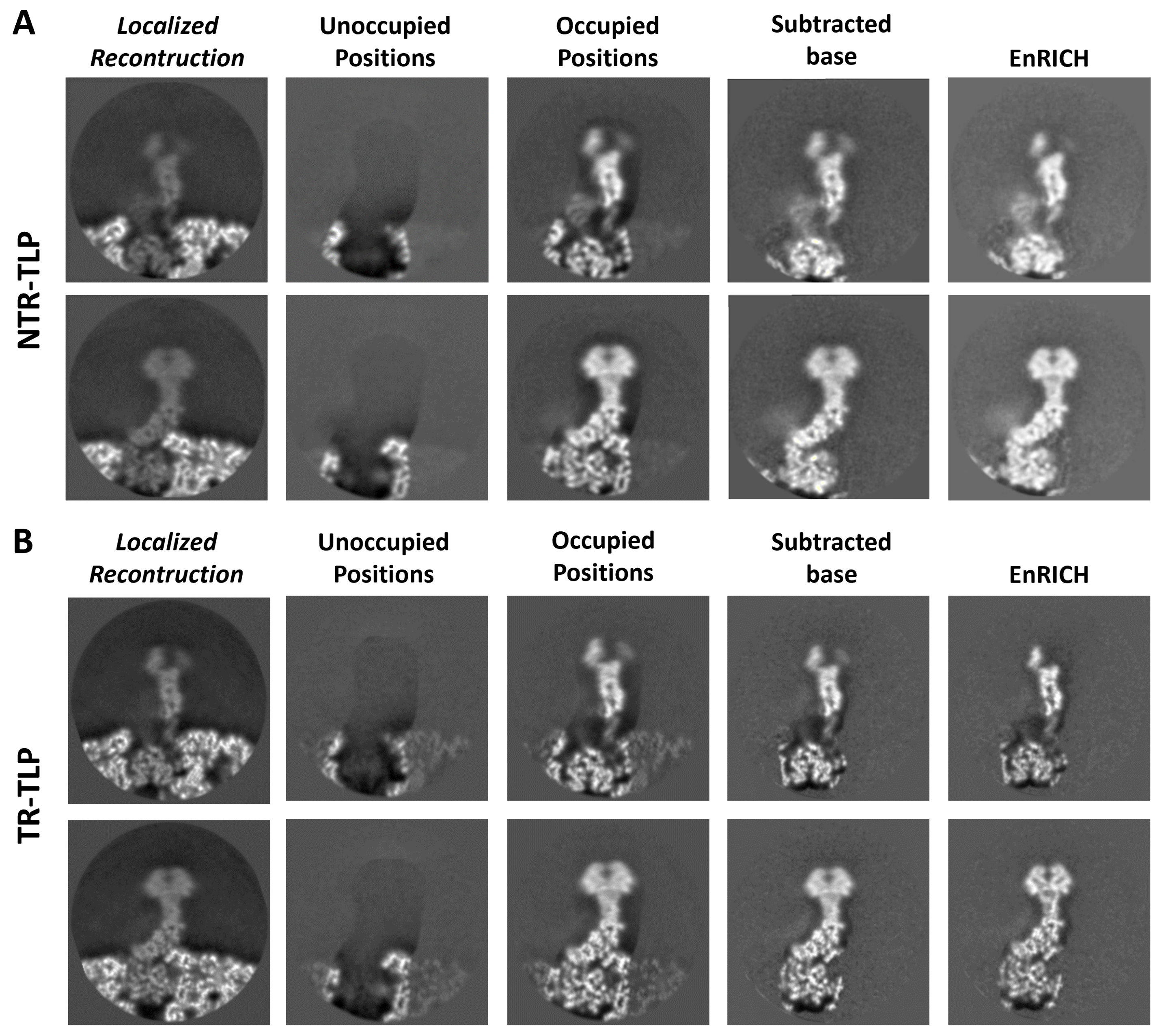

Supplement: S6 Fig — 1.34 Å thick cross sections of the maps obtained at the different stages of refinement of the VP4 NTR and TR subparticles. The panels show sections of each 3D map parallel to the central section of the maps and offset by 14.7Å (top panels) and 6.7Å (bottom panels). (TIF) [file ppat.1013063.s006.tif]

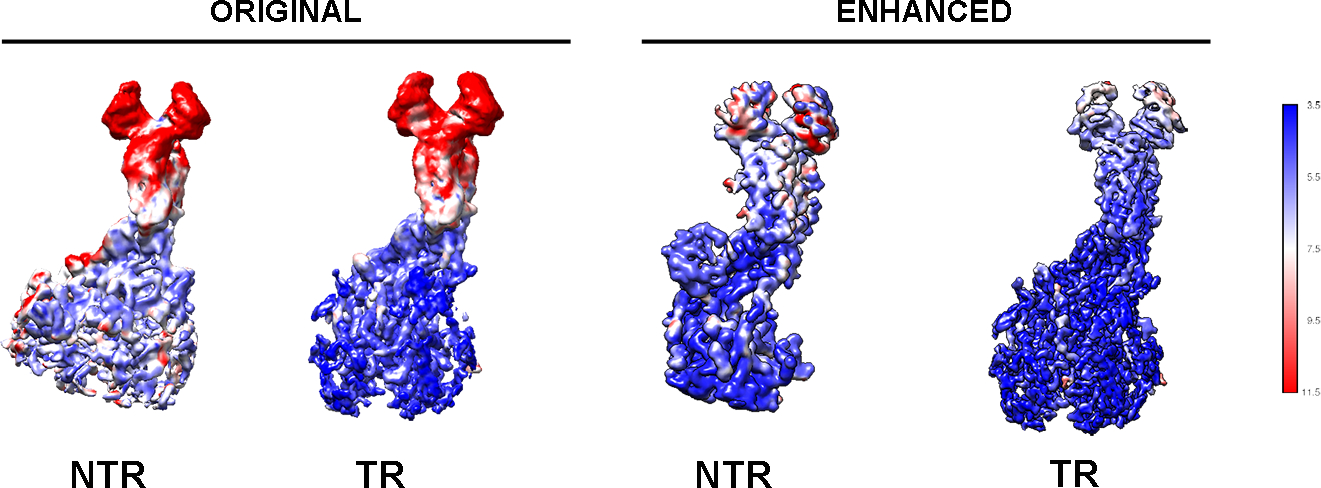

Supplement: S7 Fig — Left panel: Local resolution maps of the original reconstructions of the NTR (left) and TR (right) spikes, prior to 3D classification and signal enhancement. Right panel: Local resolution maps of the final enhanced reconstructions of the NTR (left) and TR (right) spikes following focused classification and EnRICH-based signal optimization. Resolutions are color-coded from blue (higher resolution) to red (lower resolution), as indicated by the scale bar. (TIF) [file ppat.1013063.s007.tif]

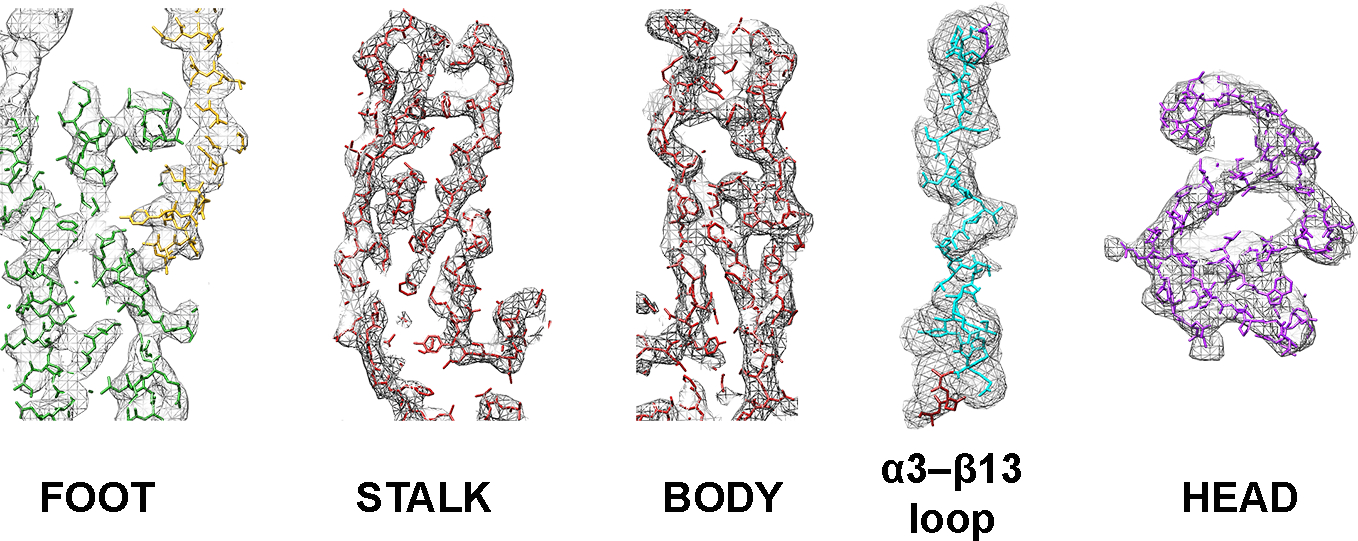

Supplement: S8 Fig — Examples of atomic model fitting into the cryo-EM density are shown for key structural regions of the TR spike: the foot, stalk, body, and head domains. The α3–β13 loop is shown from the NTR spike. Each panel displays the model overlaid on the corresponding enhanced map, illustrating the quality of the fit and interpretability in each region. (TIF) [file ppat.1013063.s008.tif]

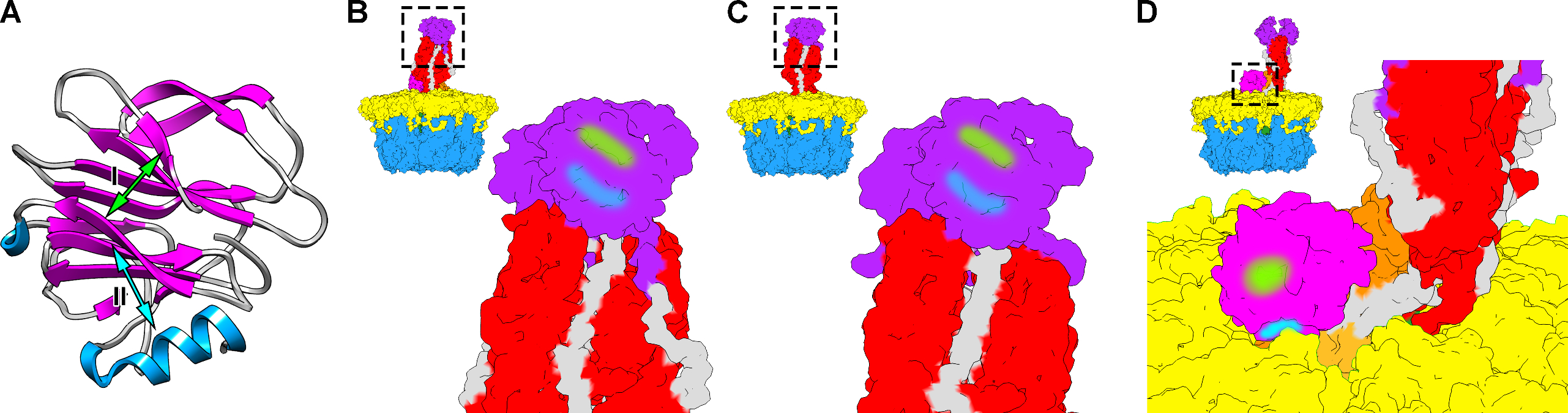

Supplement: S9 Fig — Representation of the VP8* domain of SA11 (PDB 1KQR) coloured according to its secondary structure. The glycan binding sites located in the cleft between the β-sheets (I) and adjacent to it (II) are indicated. (B-D) Accessibility of sites I (green line) and II (blue line) in the head (B, C) and stem (D) lectin domains of the NTR (B, D) and TR (C) spike. (TIF) [file ppat.1013063.s009.tif]

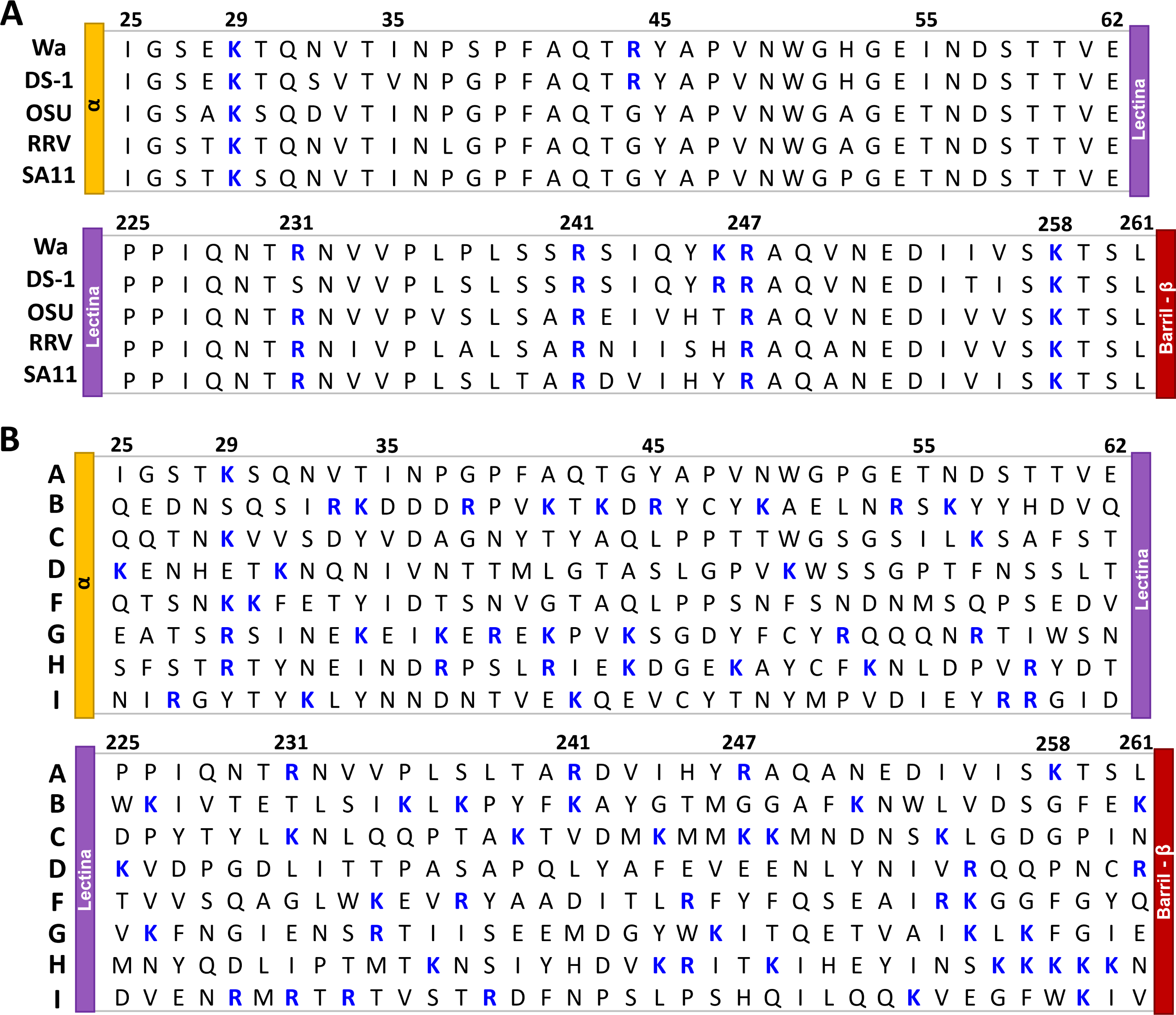

Supplement: S10 Fig — (A) Sequences corresponding to the α2- β1 loop (between the α and lectin domains, residues 25–35). (B) Sequences corresponding to the α3 − β14 loop (between the lectin and β-barrel domains of the spike body, residues 225–261). The sequences shown correspond to representative strains of the RVA species (top panel) and to the reference strain of the different RV species (bottom panel). Residues susceptible to being cut by trypsin, lysine (K) and arginine (R), are marked in blue. All aa are presented with the single letter code. (TIF) [file ppat.1013063.s010.tif]
