# Supplementary material for: Structural determinants of rotavirus proteolytic activation
Source: PLoS Pathog. 2025 Aug 12;21(8):e1013063. doi: 10.1371/journal.ppat.1013063 (PMC12364327; doi:10.1371/journal.ppat.1013063)
Supplement: S1 Table — (DOCX) [file ppat.1013063.s013.docx]

**Supplementary table**

**Table S1. Cryo-EM data collection and model statistics**

|  | **NTR-TLP** | **TR-TLP** |
| --- | --- | --- |
| **Data collection and processing** |  |  |
| Microscope | FEI Titan Krios | FEI Titan Krios |
| Detector | Falcon II | Falcon III |
| Magnification | 59000 | 58000 |
| Voltage (kV) | 300 | 300 |
| Electron exposure (e^-^/Å^2^) | 42.0 | 39.9 |
| Exposure per frame (e^-^/Å^2^) | 1.68 | 1.33 |
| Defocus range (µm) | -0.75, -3.0 | -0.75, -3.0 |
| Pixel size (Å/píxel) | 1.34 | 1.43 |
| Micrographs collected (no.) | 1465 | 2368 |
| Initial particles (no.) | 11221 | 28427 |
| Final particles (no.) | 10815 | 22394 |
| Symmetry imposed | I2 | I2 |
| Map resolution (Å) | 3.40 Å | 3.48 Å |
| FSC threshold | 0.143 | 0.143 |
| Map resolution range with spikes (Å) | 3.00 – 4.20 | 3.01 - 4.06 |
| Map resolution range without spikes (Å) | 3.00 – 13.50 | 3.01 - 13.50 |
| **Refinement** |  |  |
| Model resolution (Å) | 3.4 | 3.5 |
| FSC threshold | 0.5 | 0.5 |
| Mask correlation coefficient | 0.79 | 0.84 |
| Map sharpening B factor (Å^2^) | -135,6 | -160 |
| Cros-correlación (CC) | 0.77 | 0.83 |
| ***Model composition*** |  |  |
| Non-hydrogen atoms | 82097 | 82170 |
| Protein residues | 10264 | 10273 |
| Zn+ | 5 | 5 |
| NAG | 10 | 10 |
| Ca^2+^ | 26 | 26 |
| ***ADP (B-factors)*** |  |  |
| min | 56,5 | 63,12 |
| max | 119,74 | 265,35 |
| mean | 83,32 | 102,17 |
| ***R.m.s. deviations*** |  |  |
| Bond lengths (Å) | 0.006 | 0,008 |
| Bond angles (˚) | 0,918 | 0,695 |
| ***Validation*** |  |  |
| MolProbity score | 1.77 | 1.59 |
| Clashscore | 7.43 | 4.51 |
| Rotamer outliers (%) | 0.12 | 0.28 |
| ***Ramachandran plot*** |  |  |
| Favored (%) | 94.76 | 94.77 |
| Allowed (%) | 5.18 | 5.16 |
| Outliers (%) | 0.06 | 0.07 |
