# Supplementary material for: Structural determinants of rotavirus proteolytic activation
Source: PLoS Pathog. 2025 Aug 12;21(8):e1013063. doi: 10.1371/journal.ppat.1013063 (PMC12364327; doi:10.1371/journal.ppat.1013063)
Supplement: S2 Table — (DOCX) [file ppat.1013063.s014.docx]

**Supplementary table**

**Table S2. Accession numbers of used VP4 segments for sequence alignment.**

| **RV specie** | **Strain** | **Accession number** |
| --- | --- | --- |
| A | SA-C4111 | AIC34758.1 |
| A | DS-1 | AEG25325.1 |
| A | Wa | P11193.3 |
| A | OSU_C5111 | AIC34770.1 |
| A | RRV | P12473.2 |
| B | Bang373 | YP_008126845.1 |
| C | Bristol | CAA55958.1 |
| D | 05V0049 | YP_003896048.1 |
| F | 03V0568 | YP_008145319.1 |
| G | 03V0567 | YP_008136232.1 |
| H | J19 | YP_392492.1 |
| I | KE135 | YP_009130677.1 |
