# Supplementary material for: Structural determinants of rotavirus proteolytic activation
Source: PLoS Pathog. 2025 Aug 12;21(8):e1013063. doi: 10.1371/journal.ppat.1013063 (PMC12364327; doi:10.1371/journal.ppat.1013063)
Supplement: S3 Table — (DOCX) [file ppat.1013063.s015.docx]

**Supplementary table**

**Table S3. Atomic coordinates and EM maps accession codes**

|  | Protein Data Bank (PDB) accession code | Electron Microscopy Data Bank (EMDB) code |
| --- | --- | --- |
| NTR-TLP | 8OLB | EMD-16954 |
| NTR spike | 8OLE | EMD-16956 |
| TR-TLP | 8OLC | EMD-16955 |
| TR spike | 8QTZ | EMD-18655 |
